# Weekend effect on 30-day mortality for ischemic and hemorrhagic stroke analyzed using severity index and staffing level

**DOI:** 10.1371/journal.pone.0283491

**Published:** 2023-06-22

**Authors:** Seung Bin Kim, Bo Mi Lee, Joo Won Park, Mi Young Kwak, Won Mo Jang

**Affiliations:** 1 Interdepartment of Critical Care Medicine, Seoul Metropolitan Government-Seoul National University Boramae Medical Center, Seoul, Republic of Korea; 2 HIRA Research Institute, Health Insurance Review & Assessment Service, Wonju, Republic of Korea; 3 Center for Public Healthcare, National Medical Center, Seoul, Republic of Korea; 4 Department of Public Health and Community Medicine, Seoul Metropolitan Government-Seoul National University Boramae Medical Center, Seoul, Republic of Korea; 5 Department of Health Policy and Management, Seoul National University College of Medicine, Seoul, Republic of Korea; Stanford University School of Medicine, UNITED STATES

## Abstract

**Background and purpose:**

Previous studies on the weekend effect—a phenomenon where stroke outcomes differ depending on whether the stroke occurred on a weekend—mostly targeted ischemic stroke and showed inconsistent results. Thus, we investigated the weekend effect on 30-day mortality in patients with ischemic or hemorrhagic stroke considering the confounding effect of stroke severity and staffing level.

**Methods:**

We retrospectively analyzed data of patients hospitalized for ischemic or hemorrhagic stroke between January 1, 2015, and December 31, 2018, which were extracted from the claims database of the National Health Insurance System and the Medical Resource Report by the Health Insurance Review & Assessment Service. The primary outcome measure was 30-day all-cause mortality.

**Results:**

In total, 278,632 patients were included, among whom 84,240 and 194,392 had a hemorrhagic and ischemic stroke, respectively, with 25.8% and 25.1% of patients, respectively, being hospitalized during the weekend. Patients admitted on weekends had significantly higher 30-day mortality rates (hemorrhagic stroke 16.84%>15.55%, p<0.0001; ischemic stroke 5.06%>4.92%, p<0.0001). However, in the multi-level logistic regression analysis adjusted for case-mix, pre-hospital, and hospital level factors, the weekend effect remained consistent in patients with hemorrhagic stroke (odds ratio [OR] 1.05, 95% confidence interval [CI] 1.00–1.10), while the association was no longer evident in patients with ischemic stroke (OR 1.01, 95% CI 0.96–1.06).

**Conclusions:**

Weekend admission for hemorrhagic stroke was significantly associated with a higher mortality rate after adjusting for confounding factors. Further studies are required to understand factors contributing to mortality during weekend admission.

## Introduction

Many studies have shown that the risk of poor clinical outcomes might be higher for patients admitted on weekends than for those admitted on weekdays, a phenomenon called the “weekend effect” [1–5]. In acute stroke management, onset-to-treatment time is critical for both ischemic [3, 6] and hemorrhagic [4, 7] stroke. Since acute stroke can occur at any time, efficient stroke care should always be provided; one system is the “24/7/365 (hours per day/days per week/days per year)” emergency system [8]. However, considerable variations exist in the availability of healthcare resources for stroke treatment [9], affecting the clinical outcomes of patients.

Several systematic reviews and meta-analyses have been recently performed in an attempt to summarize studies on the weekend effect [6, 10]. One study suggested factors related to service provision inside and outside the hospital and case-mix factors that may contribute to or modify the weekend effect [11]. In-hospital factors include lower staffing levels during weekends [2], delayed assessment and management, fewer ward rounds [12], and disparities in resources and expertise [3]. Pre-hospital factors include the timeliness of patient referral and the availability of ambulance service [11]. Case-mix factors include patient characteristics and stroke severity.

Most studies investigating weekend effects were conducted on either ischemic stroke [3, 6, 13] or both types of stroke [7, 14, 15]. However, the results varied. Some studies showed no association between weekend admissions and mortality after adjusting for case-mix factors [8, 14, 16, 17]. In contrast, one study found that hemorrhagic stroke patients admitted on weekends had significantly higher in-hospital mortality rates after adjusting for patient characteristics, including comorbidities [7]. Various studies highlighted the unavailability of proper severity-of-illness measures to accurately adjust for the effect of case-mix factors as a major limitation [14]. This limitation, commonly observed in claims-based stroke studies, is particularly relevant, as it is a major determinant of stroke outcomes [18, 19]. Ideally, stroke severity should be evaluated using clinical neurological scales such as the National Institutes of Health Stroke Scale (NIHSS). However, the claims-based stroke severity index (SSI) can also be used as a proxy to measure stroke severity [20].

A weekend effect was not observed in a large cohort of patients with ischemic stroke treated at a stroke center, which was designated by the Brain Attack Coalition. This may be attributed to the 24/7/365 access to stroke specialists, nurses handling stroke cases, and the organized system for delivering care available at stroke centers [16]. However, previous studies did not identify the timeliness of patient transfers to the hospital. In this study, we attempted to adjust for factors affecting healthcare delivery at a pre-hospital stage through variables involved in direct contact with and transferring to severe emergency centers.

Several previous studies indicated that the weekend effect remains unclear even after adjusting for case-mix factors and that the findings are insufficient, as factors influencing emergency delivery systems were not considered. In this study, we attempted to examine the effect of weekend admission on 30-day mortality of patients with ischemic and hemorrhagic stroke after adjusting for explanatory factors, classified into case-mix, pre-hospital level, and hospital level factors.

## Methods

### Data source

We retrospectively analyzed data extracted from the National Health Insurance System (NHIS) claims database from 2015 to 2018 and the Medical Resource Report by the Health Insurance Review & Assessment Service (HIRA) in 2018. Since almost all payments were based on a fee-for-service system, the NHIS claims database contains specific disease codes and all necessary data for reimbursement. These data include patient socio-demographic information, such as sex, age, health insurance type, residential area, comorbid diseases, diagnostic tests, procedures, operations and prescriptions, and outcomes (including deaths). We constructed the dataset by adding hospital characteristics (e.g., staff and facility) from the Medical Resource Report (HIRA). This study was reviewed and approved by the Institutional Review Board of Seoul National University College of Medicine (IRB No. 07-2021-8). Informed consent was waived owing to the retrospective nature of the study.

### Study population

We included all hospitalized ischemic and hemorrhagic stroke patients who had been admitted to the emergency department between January 1, 2015, and December 31, 2018. We excluded patients aged <20 years and those admitted to clinics using the same cutoffs as previously described [13]. Stroke was diagnosed using the International Classification of Disease 10^th^ Revision (ICD-10) primary diagnosis codes: (1) hemorrhagic stroke (ICD-10 codes I60–I62) and (2) ischemic stroke (ICD-10 code I63) [21]. A single admission episode was defined as hospitalization and discharge during a single day in the same hospital, as multiple billing data could be claimed on a single day at the same hospital owing to separate monthly claims.

A total of 286,606 cases were included, and cases with missing values were excluded. Ultimately, 278,632 cases were included for analysis.

### Variables

The dependent variable was all-cause mortality within 30 days of each admission for ischemic/hemorrhagic stroke [13, 17]. We defined the admission date of the first hospitalization for an episode as the index date, and if the date of death was included within 30 days of the index date, the case was classified to have mortality within 30 days. Weekend admission was investigated by determining whether patients with ischemic/hemorrhagic stroke were admitted to the emergency department on a Saturday or Sunday. Patient and hospital characteristics were classified as covariates.

We classified case-mix and service provision factors as covariates at the in- and pre-hospital levels [11]. Case-mix factors included age (continuous), sex, health insurance type (national health insurance or medical aid), income level quartile (Q1–Q4), SSI, and interventions (medication, procedures, operations) provided to the patient (yes/no) (S1 and S2 Tables) [22–24].

A severe emergency center included a tertiary hospital, regional or local emergency center (>500 hospital beds), or regional cardiocerebrovascular center. The type of contact with a severe emergency center was classified as direct or transferring. Direct contact indicates that a severe emergency patient who required treatment at a center-level institution was sent directly to a severe emergency center. In contrast, transferring means that a severe emergency patient first went to a local emergency medical agency or an unqualified institution.

We classified the type of hospital based on the final medical treatment institution if the initial assessment hospital differs from the final treatment hospital within a single day. Hospital level service provision included hospital type (tertiary hospital, general hospital, hospital); bed size (<300 beds, 300–500 beds, 500–1,000 beds, >1,000 beds); ownership (public/private); stroke center (yes/no), designated by the Korea Stroke Society as an institution equipped with proper facilities, staffing, and tools to properly provide core treatment for patients with stroke; intervention volume indicating whether the hospital implemented more than the threshold of annual intervention (yes/no) (S3 Table) [20, 25–33]; number of physicians including neurologists, neurosurgeons, emergency medical personnel, and radiologists (25^th^ quartile: 25, 50^th^ quartile: 32, 75^th^ quartile: 42); and number of nurses (25^th^ quartile: 426, 50^th^ quartile: 724, 75^th^ quartile: 966). We conducted the pre-hospital level analysis based on the 70 units of the catchment area classified by the Ministry of Health & Welfare (S1 Fig, S4 Table).

### Stroke severity index

We used the SSI published in Taiwan [34, 35] as a proxy for the NIHSS in the model. The SSI was validated by demonstrating a close correlation between SSI results and actual stroke severity assessed using the NIHSS. The SSI comprises seven claim items: airway suctioning, bacterial sensitivity test, general ward stay, intensive care unit stay, nasogastric intubation, osmotherapy, and urinary catheterization [34]. We used the criteria developed by customizing the coefficient values of each of the seven parameters to the Korean HIRA database (S5 Table). The SSI was obtained using the regression coefficients estimated from a multiple linear regression equation in a previous study (S6 Table) [20]. The SSI was validated based on the NIHSS related to the ischemic stroke evaluation index; however, its validity was also confirmed for hemorrhagic stroke [36].

### Statistical analysis

Continuous variables are summarized as mean and standard deviation, and categorical variables are summarized as frequencies and percentages. Variables were compared between groups using Student’s t-test for continuous variables and the Chi-square tests for categorical variables. We performed hierarchical logistic regression analysis using multi-level models with the generalized linear mixed model (GLIMMIX) procedure at three levels, comprising case-mix (patient level), pre-hospital level (contact type), and hospital level variables. In this analysis, we examined their association with weekend admission and 30-day mortality after admission. All statistical analyses were performed using SAS statistical software version 9.3 (SAS Institute Inc., Cary, NC, USA). All p-values were two-sided and considered significant at <0.05.

## Results

### Characteristics of patients admitted for stroke

In our study, 84,240 and 194,392 patients were diagnosed with hemorrhagic and ischemic stroke, respectively (Table 1). In the hemorrhagic stroke group, the mortality rate for weekend admission was higher than that for weekday admission (16.84%>15.55%; p<0.0001). The rate of female patients (50.90%>49.21%; p<0.0001) and the rate of patients who had received interventions (procedures 14.86%>14.13%; operations 16.75%>15.67%, p<0.0001) were higher in weekend admission than in weekday admission. Regarding the type of contact with the severe emergency center, the rate of direct type was higher in weekend admission than in weekday admission (81.32%>79.81%, p<0.0001). Additionally, the mean SSI score in patients admitted on weekends was higher than that in patients admitted on weekdays (11.13>10.77, p<0.0001).

**Table 1:** Characteristics of the study population.

Variables			Hemorrhagic stroke (N = 84,240)			Ischemic stroke (N = 194,392)		
			Weekend	Weekday	p-value	Weekend	Weekday	p-value
			(N = 21,721)	(N = 62,519)		(N = 50,743)	(N = 143,649)	
Death within 30 days after admission								
Died			3,658 (16.84)	9,721 (15.55)	<0.0001	2,569 (5.06)	7,073 (4.92)	<0.0001
Alive			18,063 (83.16)	52,798 (84.45)		48,174 (94.94)	136,576 (95.08)	
**Case-mix**								
	Sex							
	Male		10,666 (49.10)	31,751 (50.79)	<0.0001	28,815 (56.79)	82,306 (57.30)	<0.0001
	Female		11,055 (50.90)	30,768 (49.21)		21,928 (43.21)	61,343 (42.70)	
	Age (mean, SD)		69.18 (15.30)	63.19 (14.55)	<0.0001	78.81 (11.11)	70.65 (12.86)	<0.0001
	Income level							
	Health insurance	1^st^ quartile	4,186 (19.27)	11,899 (19.03)	<0.0001	8,997 (17.73)	25,475 (17.73)	<0.0001
		2^nd^ quartile	4,101 (18.88)	11,847 (18.95)		8,236 (16.23)	23,583 (16.42)	
		3^rd^ quartile	4,891 (22.52)	14,155 (22.64)		10,814 (21.31)	30,462 (21.21)	
		4^th^ quartile	7,047 (32.44)	20,259 (32.40)		18,680 (36.81)	52,094 (36.26)	
	Medical aid		1,496 (6.89)	4,359 (6.97)		4,016 (7.91)	12,035 (8.38)	
	SSI score (m, SD)		11.13 (4.61)	10.77 (4.65)	<0.0001	6.08 (4.00)	6.03 (3.96)	<0.0001
	Intervention-Medication*							
	Yes		-	-		47,610 (93.83)	134,199 (93.42)	<0.0001
	No		-	-		3,133 (6.17)	9,450 (6.58)	
	Intervention-Procedure*							
	Yes		3,227 (14.86)	8,835 (14.13)	<0.0001	4,050 (7.98)	11,513 (8.01)	<0.0001
	No		18,494 (85.14)	53,684 (85.87)		46,693 (92.02)	132,136 (91.99)	
	Intervention-Operation*							
	Yes		3,639 (16.75)	9,797 (15.67)	<0.0001	685 (1.35)	2,022 (1.41)	<0.0001
	No		18,082 (83.25)	52,722 (84.33)		50,058 (98.65)	141,627 (98.59)	
**Pre-hospitallevel**								
	Type of contact with severe emergency center							
	Direct		17,663 (81.32)	49,896 (79.81)	<0.0001	39,949 (78.73)	113,494 (79.01)	0.184
	Transferring		4,058 (18.68)	12,623 (20.19)		10,794 (21.27)	30,155 (20.99)	
**Hospital level**								
	Type							
	Tertiary hospital		10,215 (47.03)	28,988 (46.37)	0.0304	23,434 (46.18)	67,508 (47.00)	0.0039
	General hospital		11,188 (51.51)	32,477 (51.95)		26,095 (51.43)	72,636 (50.56)	
	Hospital		318 (1.46)	1,054 (1.69)		1,217 (2.40)	3,505 (2.44)	
	Bed volume							
	≤299		1,595 (7.34)	5,202 (8.32)	<0.0001	4,891 (9.64)	14,239 (9.91)	<0.0001
	300–499		2,574 (11.85)	7,530 (12.04)		6,189 (12.20)	16,548 (11.52)	
	500–999		13,063 (60.14)	37,391 (59.81)		29,008 (57.17)	81,341 (56.62)	
	≥1,000		4,489 (20.67)	12,396 (19.83)		10,655 (21.00)	31,521 (21.94)	
	Ownership							
	Public		4,547 (20.93)	12,702 (20.32)	0.0638	11,532 (22.73)	34,084 (23.73)	<0.0001
	Private		10,806 (79.09)	49,817 (79.68)		39,211 (77.27)	109,565 (76.27)	
	Stroke center							
	Yes		9,131 (42.04)	26,931 (43.08)	0.0077	22,395 (44.13)	63,480 (44.19)	0.8245
	No		12,590 (57.96)	35,588 (56.92)		28,348 (55.87)	80,169 (55.81)	
	Intervention volume							
	High		19,853 (91.40)	56,392 (90.20)	<0.0001	44,580(87.85)	125,933(87.67)	0.2691
	Low		1,868 (8.60)	6,127 (9.80)		6,163(12.15)	17,716 (12.33)	
	Number of physicians							
	1^st^ quartile		5,032 (23.17)	15,171 (24.27)	0.0099	13,034 (25.69)	35,730 (24.87)	0.0001
	2^nd^ quartile		5,200 (23.94)	14,599 (23.35)		12,056 (23.76)	33,985 (23.66)	
	3^rd^ quartile		5,732 (26.39)	16,298 (26.07)		12,570 (24.77)	35,642 (24.81)	
	4^th^ quartile		5,757 (26.50)	16,451 (26.31)		13,083 (25.78)	38,292 (26.66)	
	Number of nurses							
	1^st^ quartile		4,887 (22.50)	14,774 (23.63)	0.0086	12,936 (25.49)	35,761 (24.89)	<0.0001
	2^nd^ quartile		5,707 (26.27)	16,248 (25.99)		12,485 (24.60)	34,311 (23.89)	
	3^rd^ quartile		5,630 (25.92)	15,910 (25.45)		12,709 (25.05)	36,400 (25.34)	
	4^th^ quartile		5,497 (25.31)	15,587 (24.93)		12,613 (24.86)	37,177 (25.88)	

SD, standard deviation; SSI, stroke severity index.

p<0.05 calculated using t-test and χ^2^ test.

* S1 and S2 Tables present definitions of interventions (medication, procedures, and operations) in ischemic/hemorrhagic stroke.

The results for ischemic stroke were similar to those for hemorrhagic stroke. In the ischemic stroke group, the mortality rate for weekend admission was higher than that for weekday admission (5.06%>4.92%; p<0.0001). However, regarding the type of contact with the severe emergency center, the rate of direct type was lower on weekend admission than in weekday admission, although not significantly different (78.73%<79.01%, p = 0.184).

The characteristics of patients with 30-day mortality after admission are presented in Table 2.

**Table 2:** Characteristics of patients with 30-day mortality after admission.

Variable			Hemorrhagic stroke (N = 84,240)			Ischemic stroke (N = 194,392)		
			Died	Alive	p-value	Died	Alive	p-value
			(N = 13,379)	(N = 70,861)		(N = 9,642)	(N = 184,750)	
Hospitalization								
Weekday			9,721 (72.66)	52,798 (74.51)	<0.0001	7,073 (73.36)	136,576 (73.92)	0.2152
Weekend			3,658 (27.34)	18,063 (25.49)		2,569 (26.64)	48,174 (26.08)	
**Case-mix**								
	Sex							
	Male		6,464 (48.31)	35,953 (50.74)	<0.0001	4,447 (46.12)	106,674 (57.74)	<0.0001
	Female		6,915 (51.69)	34,908 (49.26)		5,195 (53.88)	78,076 (42.26)	
	Age		69.18 (15.3)	63.19 (14.55)	<0.0001	78.81 (11.11)	70.65 (12.86)	<0.0001
	Income level							
	Health insurance	1^st^ quartile	2,407 (17.99)	13,678 (19.30)	<0.0001	1,712 (17.76)	32,760 (17.73)	<0.0001
		2^nd^ quartile	2,343 (17.51)	13,605 (19.20)		1,417 (14.70)	30,402 (16.46)	
		3^rd^ quartile	2,877 (21.50)	16,169 (22.82)		1,873 (19.43)	39,403 (21.33)	
		4^th^ quartile	4,465 (33.37)	22,841 (32.23)		3,631 (37.66)	67,143 (36.34)	
	Medical aid		1,287 (9.62)	4,568 (6.45)		1,009 (10.46)	15,042 (8.14)	
	SSI score		14.4 (3.55)	10.2 (4.52)	<0.0001	12.18 (4.64)	5.72 (3.66)	<0.0001
	Intervention-Medication*							
	Yes		-	-		8,053 (83.52)	173,756 (94.05)	<0.0001
	No		-	-		1,589 (16.48)	10,994 (5.95)	
	Intervention-Procedure*							
	Yes		1,465 (10.95)	10,597 (14.95)	<0.0001	1,532 (15.89)	14,031 (7.59)	<0.0001
	No		11,914 (89.05)	60,264 (85.05)		8,110 (84.11)	170,719 (92.41)	
	Intervention-Operation*							
	Yes		2,026 (15.14)	11,410 (16.10)	0.0052	425 (4.41)	2,282 (1.24)	<0.0001
	No		11,353 (84.86)	59,451 (83.90)		9,217 (95.59)	182,468 (98.76)	
**Pre-hospital level**								
	Type of contact with severe emergency center							
	Direct		10,375 (77.55)	57,184 (80.70)	<0.0001	7,208 (74.76)	146,235 (79.15)	<0.0001
	Transferring		3,004 (22.45)	13,677 (19.30)		2,434 (25.24)	38,515 (20.85)	
**Hospital level**								
	Type							
	Tertiary hospital		5,794 (43.34)	33,409 (47.15)	<0.0001	4,093 (42.45)	86,846 (47.01)	<0.0001
	General hospital		7,256 (54.23)	36,409 (51.38)		5,230 (54.24)	93,501 (50.61)	
	Hospital		329 (2.46)	1,043 (1.47)		319 (3.31)	4,403 (2.38)	
	Bed volume							
	≤299		1,276 (9.54)	5,521 (7.79)	<0.0001	1,156 (11.99)	17,974 (9.73)	<0.0001
	300–499		1,787 (13.36)	8,317 (11.74)		1,322 (13.71)	21,415 (11.59)	
	500–999		8,158 (60.98)	42,296 (59.69)		5,459 (56.62)	104,890 (55.77)	
	≥1000		2,158 (16.13)	14,727 (20.78)		1,705 (17.68)	40,471 (21.91)	
	Ownership							
	Public		2,573 (19.23)	14,670 (20.70)	0.1785	2,229 (23.12)	43,387 (23.48)	0.4069
	Private		10,806 (80.77)	56,191 (79.30)		7,413 (76.88)	141,363 (76.52)	
	Stroke center							
	Yes		7,027 (52.52)	41,151 (58.07)	<0.0001	4,906 (50.88)	103,611 (56.08)	<0.0001
	No		6,352 (47.48)	29,710 (41.93)		4,736 (49.12)	81,139 (43.92)	
	Intervention volume							
	High		11,747 (87.80)	64,498 (91.02)	<0.0001	8,123 (84.25)	162,390 (87.90)	<0.0001
	Low		1,632 (12.20)	6,363 (8.98)		1,519 (15.75)	22,360 (12.10)	
	Number of physicians							
	1^st^ quartile		3,677 (27.48)	16,526 (23.32)	<0.0001	2,915 (30.23)	45,849 (24.82)	<0.0001
	2^nd^ quartile		3,260 (24.37)	16,539 (23.34)		2,372 (24.60)	43,669 (23.64)	
	3^rd^ quartile		3,506 (26.21)	18,524 (26.14)		2,262 (23.46)	45,950 (24.87)	
	4^th^ quartile		2,936 (21.94)	19,272 (27.20)		2,093 (21.71)	49,282 (26.67)	
	Number of nurses							
	1^st^ quartile		3,560 (26.61)	16,101 (22.72)	<0.0001	2,878 (29.85)	45,819 (24.80)	<0.0001
	2^nd^ quartile		3,542 (26.47)	18,413 (25.98)		2,316 (24.02)	44,480 (24.08)	
	3^rd^ quartile		3,418 (25.55)	18,122 (25.57)		2,378 (24.66)	46,731 (25.29)	
	4^th^ quartile		2,859 (21.37)	18,225 (25.72)		2,070 (21.47)	47,720 (25.83)	

SSI, stroke severity index.

Data are presented as mean±standard deviation or n (%). p<0.05 calculated using t-test and χ^2^ test.

* S1 and S2 Tables present definitions of interventions (medication, procedures, and operations) in ischemic/hemorrhagic stroke.

### Case-mix variables in hemorrhagic stroke

The rate of female patients who died was higher than that among those who survived (51.69%>49.26%, p<0.0001). The average age of those who died was higher than that of the survivors (69.18 years>63.19 years, p<0.0001). The average SSI score of patients who died was higher than that of the survivors (14.4>10.2, p<0.0001). The rate of patients who had not received intervention was higher among those who died than among the survivors (procedures 89.05%>85.05%, p<0.0001; operation 84.86%>83.90%, p = 0.0052).

### Case-mix variables in ischemic stroke

The rate of female patients who died was higher than that among those who survived (53.88%>42.26%, p<0.0001). The average age of patients who died was higher than that of the survivors (78.81>70.65 years, p<0.0001). The average SSI score of patients who died was higher than that of the survivors (12.18>5.72, p<0.0001). The rate of patients who had not received medication was higher among those who died than among the survivors (16.48%>5.95%, p<0.0001); however, the rate of patients who had not received intervention (procedures and operation) was lower among those who died than among the survivors (procedures 84.11%<92.41%, p<0.0001; operation 95.59%<98.76%, p<0.0001).

### Pre-hospital level variables

Regarding the type of contact with the severe emergency center, the rate for the transferring type among those who died was higher than among those who survived (hemorrhagic stroke 22.45%>19.30%; ischemic stroke 25.24%>20.85%, p<0.0001).

### Hospital level variables in hemorrhagic stroke

The rate of tertiary hospitals was lower among those who died than among the survivors (43.34%<47.15%, p<0.0001). Moreover, the rate of hospitals with more than 1000 beds (16.13%<20.78%, p<0.0001) and the rate of hospitals with a stroke center (52.52%<58.07%, p<0.0001) were lower in those who died than in those who survived. The rate of hospitals with a high intervention volume was lower in those who died than in those who survived (87.80%<91.02%, p<0.0001). Regarding staff numbers, the fewer the number of physicians, the higher the mortality rate (p<0.0001). This trend was also observed for the number of nurses (p<0.0001).

### Hospital level variables in ischemic stroke

The rate of tertiary hospitals was lower among those who died than among the survivors (42.45%<47.01%, p<0.0001). Moreover, the rate of hospitals with more than 1000 beds (17.68%<21.91%, p<0.0001) and the rate of hospitals with a stroke center (50.88%<56.08%, p<0.0001) were lower in those who died than among the survivors. The rate of hospitals with a high intervention volume was lower in those who died than among the survivors (84.25%<87.90%, p<0.0001). Regarding staff numbers, the fewer the number of physicians and nurses, the higher the mortality rate (p<0.0001 for both professionals).

### Multi-level logistic regression analysis in hemorrhagic and ischemic stroke

#### Mortality risk in patients with hemorrhagic stroke

Patients admitted on weekends had a significantly higher 30-day mortality risk than those admitted on weekdays (odds ratio [OR] 1.05, 95% confidence interval [CI] 1.00–1.10). Regarding the case-mix variables, older age (OR 1.02; 95% CI 1.02–1.02), medical aid (ref = quartile 4 of health insurance; OR 1.16; 95% CI 1.06–1.27), and a higher SSI score (OR 1.29; 95% CI 1.28–1.30) were associated with a higher mortality risk. The mortality risk of patients who did not receive intervention was higher than that of patients who received the intervention (OR 1.94, 95% CI 1.80–2.09). Furthermore, patients who did not undergo an operation had a higher mortality risk (OR 2.08, 95% CI 1.94–2.24) than did patients who underwent an operation (Table 3).

**Table 3:** Multi-level logistic regression analysis of 30-day mortality.

Variable			Hemorrhagic stroke			Ischemic stroke		
			OR	95% CI	p-value	OR	95% CI	p-value
Hospitalization								
Weekend			1.05	1.00–1.10†	0.0357	1.01	0.96–1.06	0.7315
Weekday			1.00			1.00		
**Case-mix**								
	Sex							
	Male		1.00			1.00		
	Female		0.97	0.92–1.02	0.2424	1.09	0.99–1.10	0.1058
	Age		1.02	1.02–1.02†	<0.0001	1.04	1.04–1.04†	<0.0001
	Income level							
	Health insurance	1^st^ quartile	0.95	0.90–1.00	0.0468	1.09	1.02–1.16†	0.0071
		2^nd^ quartile	1.00	0.93–1.06	0.8905	1.07	0.99–1.15	0.071
		3^rd^ quartile	0.99	0.93–1.04	0.6442	1.03	0.97–1.10	0.3916
		4^th^ quartile	1.00			1.00		
	Medical aid		1.16	1.06–1.27†	0.001	0.97	0.90–1.05	0.4277
	SSI score		1.29	1.28–1.30†	<0.0001	1.35	1.34–1.36†	<0.0001
	Intervention-Medication*							
	Yes		-	-		1.00		
	No		-	-		3.84	3.48–4.23†	<0.0001
	Intervention-Procedure*							
	Yes		1.00			1.00		
	No		1.94	1.80–2.09†	<0.0001	1.13	1.04–1.24†	0.0048
	Intervention-Operation*							
	Yes		1.00			1.00		
	No		2.08	1.94–2.24†	<0.0001	1.43	1.27–1.61†	<0.0001
**Pre-hospital level**								
	Type of contact with severe emergency center							
	Direct		1.00			1.00		
	Transferring		1.06	0.84–1.33	0.6528	1.02	0.70–1.49	0.9334
**Hospital level**								
	Type							
	Tertiary hospital		1.00			1.00		
	General hospital		0.95	0.88–1.04	0.2775	0.94	0.82–1.07	0.3317
	Hospital		2.15	1.69–2.73†	<0.0001	1.55	1.22–1.97†	0.0004
	Bed volume							
	≤299		1.15	0.85–1.55	0.3717	0.86	0.54–1.36	0.5086
	300–499		1.17	0.89–1.54	0.2603	0.94	0.60–1.48	0.8000
	500–999		1.18	1.03–1.35†	0.0154	1.09	0.93–1.27	0.3080
	≥1000		1.00			1.00		
	Ownership							
	Public		1.00			1.00		
	Private		0.99	0.90–1.10	0.9162	1.03	0.95–1.11	0.5139
	Stroke center							
	Yes		1.00			1.00		
	No		1.03	0.97–1.09	0.3533	0.93	0.87–1.00	0.0527
	Intervention volume							
	High		1.00			1.00		
	Low		1.45	1.28–1.64†	<0.0001	1.30	1.09–1.56†	0.0045
	Number of physicians							
	1^st^ quartile		1.12	0.95–1.31	0.1738	1.02	0.78–1.35	0.8721
	2^nd^ quartile		1.08	0.95–1.22	0.2507	1.10	0.94–1.30	0.2467
	3^rd^ quartile		1.08	0.97–1.21	0.1707	1.05	0.923–1.19	0.4691
	4^th^ quartile		1.00			1.00		
	Number of nurses							
	1^st^ quartile		0.93	0.77–1.13	0.4717	0.93	0.77–1.11	0.4089
	2^nd^ quartile		0.97	0.87–1.09	0.635	0.94	0.8–1.07	0.3308
	3^rd^ quartile		1.04	0.93–1.15	0.495	0.89	0.80–0.99†	0.0294
	4^th^ quartile		1.00			1.00		

†p<0.05 calculated using logistic regression analysis.

OR, odds ratio; CI, confidence interval; SSI, stroke severity index.

* S1 and S2 Tables present definitions of interventions (medication, procedures, and operations) in ischemic/hemorrhagic stroke.

Regarding pre-hospital level variables, the mortality risk for the transferring type of contact with a severe emergency center was higher than that for the direct type, although not significantly different (OR 1.06, 95% CI 0.84–1.33). Additionally, the subgroup analysis showed that patients who had the transferring type of contact with a severe emergency center had higher 30-day mortality for weekend admission (OR 1.36; 95% CI 1.06–1.75, S7 Table). Regarding hospital level variables, lower-level hospitals had a higher mortality risk than tertiary hospitals (OR 2.15; 95% CI 1.69–2.73). Hospitals with a bed volume of 500–1,000 had a higher mortality risk than hospitals with ≥1,000 beds (OR 1.18; 95% CI 1.08–1.35). Hospitals with low intervention volumes had a higher mortality risk than those with high intervention volumes (OR 1.45; 95% CI 1.28–1.64).

#### Mortality risk in patients with ischemic stroke

The effect of weekend admission on mortality was not statistically significant in patients with ischemic stroke (OR 1.01, 95% CI 0.96–1.06). Regarding case-mix variables, older age (OR 1.04; 95% CI 1.04–1.04), medical aid (ref = quartile 4 of health insurance; OR 1.09; 95% CI 1.02–1.16), and a higher SSI score (OR 1.35; 95% CI 1.34–1.36) had a higher mortality risk. Patients without interventions had a higher mortality risk (medication OR 3.84, 95% CI 3.48–4.23; procedure OR 1.13, 95% CI 1.04–1.24; operation OR 1.43, 95% CI 1.27–1.61) (Table 3).

Regarding pre-hospital level variables, the mortality risk for the transferring type of contact with the severe emergency center was higher than that for the direct type, although not significantly different (OR 1.02; 95% CI 0.70–1.49). Regarding hospital level variables, lower-level hospitals had a higher mortality risk than tertiary hospitals (OR 1.55; 95% CI 1.22–1.97). Hospitals with low intervention volumes had a higher mortality risk than those with high intervention volumes (OR 1.30; 95% CI 1.09–1.56). The mortality risk regarding staffing numbers (physicians and nurses) was not statistically significant.

## Discussion

Our study explored the effect of weekend admission on 30-day mortality in patients with ischemic or hemorrhagic stroke and confirmed explanatory factors influencing the weekend effect. We found higher mortality rates for weekend admission in patients with hemorrhagic stroke, even after adjusting for case-mix and service provision in-/pre-hospital variables.

We found that hemorrhagic stroke patients admitted during weekends had a higher risk of death than those admitted on weekdays. Previous studies found that weekend admission for ischemic stroke was associated with higher 30-day all-cause mortality [3, 6, 13]. In contrast, other studies did not find significant associations between weekend admission for ischemic stroke and higher mortality [8, 16, 17, 37, 38]. When confirming the weekend effect for total stroke, the results showed that mortality was significantly higher in patients with hemorrhagic stroke admitted on weekends than in those admitted on weekdays but not in patients with ischemic stroke [7], which is consistent with our findings. However, previous studies failed to adjust for variables, including stroke severity and time from onset to arrival. Empirical evidence of the weekend effect on stroke mortality is mixed, with some studies indicating significantly higher mortality for weekend admissions and others finding no differences [14]. The presence or magnitude of the weekend effect varies based on the types of admission, case-mix factors and illness severity, geographic location, as well as contextual and methodological factors [11]. Thus, we analyzed the weekend effect by classifying confounding factors into service provision hospital level, case-mix, and service provision pre-hospital level variables.

Some studies showed that the weekend effect on mortality disappeared after adjusting for stroke severity scores, such as the NHISS and Charlson comorbidity index. The results also varied depending on the severity scale used [3, 7, 8, 13, 14, 16, 17]. This study used the SSI, a claims-based proxy for stroke severity. Patients admitted on weekends had a higher SSI score than those admitted on weekdays, whereas the number of admissions on weekends was lower than that on weekdays (S2 and S3 Figs). A negative correlation was found between the SSI score and the number of admissions by the day of the week, which is consistent with the results of previous studies [17]. This result is thought to be due to the fact that patients who had mild stroke during weekends postponed their hospital visits to a weekday, as described in a previous study [17]; this event is called the “Monday effect.” A review of previous studies [39] that analyzed the number of patients with stroke onset and hospital presentation by the day of the week corroborates this finding; there was minimal difference in the number of patients with stroke onset and hospital presentation for moderate or severe stroke, but a significant difference was observed for mild stroke. However, their results showed that the weekend effect disappeared after adjusting for SSI [17]. In our study, although there was a decrease in the effect, it remained statistically significant in the case of hemorrhagic stroke. This could be attributed to factors other than the difference in disease severity at hospitalization, which were deemed important in the weekend effect in hemorrhagic stroke. A subgroup analysis was performed on hemorrhagic stroke to analyze these additional factors, and the results are discussed below.

In our study, hospital intervention volume significantly affected mortality, although we could not find an effect of staffing level. The problem of resource allocation between weekdays and weekends has been reported, with physician volume and experience level considered to be important factors [3, 6]. However, the effect of staffing numbers was not significant as shown by the multi-level logistic regression analysis (Table 3). Reduced availability of clinical personnel on weekends may reduce the quality of care and influence outcomes following stroke [40]. Evidence from previous studies suggests that specialized stroke units, with around-the-clock availability of specialist stroke teams and rapid access to imaging and thrombolysis, reduce variations in the quality of care and outcomes throughout the week [16, 31, 37]. Our study found that intervention volume significantly affected mortality, suggesting that the availability of treatments may be more important than staffing numbers for patient outcomes.

Moreover, we found that the healthcare delivery system may influence the weekend effect on patients with hemorrhagic stroke. We conducted a subgroup analysis by dividing patients with hemorrhagic stroke who showed significant weekend effects into weekend and weekday groups after adjusting for various confounding factors (S7 Table). Among hemorrhagic stroke patients admitted on weekends, patients who had transferred with the severe emergency center had higher 30-day mortality after adjusting for the stroke severity. This suggests that patients with hemorrhagic stroke may have problems being directly admitted to the hospital on a weekend. Therefore, it is important to establish an extensive cooperative system with severe emergency centers and other medical facilities to ensure the availability of interventions and other resources, even on weekends. Reorganizing stroke care to provide 24/7 access to stroke specialists, adequate staffing of nurses handling stroke cases on weekends, and an organized system for delivering care may alleviate the weekend effect and save lives [16, 17, 41, 42].

Our study has some limitations. First, we applied the SSI after validation in Korea [20], using a study methodology in Taiwan. Further investigations are required to determine the applicability of the SSI as a proxy for stroke severity in claims databases from other healthcare systems. Second, the diagnosis accuracy of stroke could not be guaranteed from the claims data. However, we expect that the diagnostic consistency in stroke could be generally reliable, and we extracted the population at primary diagnosis [43]. Third, we did not measure other indicators of care quality, such as onset-to-treatment time, time to assessment of rehabilitation, the intensity of rehabilitation therapy, and patient education level. Finally, we investigated the hospital level characteristics such as staffing level and bed number. However, we defined a weekend as Saturday and Sunday only, and we did not include holidays if they fell on weekdays. Moreover, we could not examine variables that reflected differences in staffing level, including the number and experience of staff members during weekdays and weekends. Therefore, we could not identify the effect of differences in staffing levels on weekends.

In summary, the significance of the weekend effect on mortality was maintained in hemorrhagic stroke but not in ischemic stroke after adjusting for case-mix, hospital level, and pre-hospital level factors. Further research is required to analyze mortality in weekend and weekday holidays with more detailed variables for patient risk factors, hospital staffing level changes, and emergency delivery systems. Our results suggested that further endeavor is needed to find effective measures, including a systematic approach for mitigating the weekend effect on hemorrhagic stroke. More research is required to explore the proper outcome variable (e.g., disability occurrence) for ischemic stroke’s weekend effect.

## Supporting information

S1 Fig Rationale for using RI*CI for the consolidation of catchment areas. (TIF)

S2 Fig Thirty-day mortality after hospital admission and the mean stroke severity index score according to the day of the week. (TIF)

S3 Fig Number of admissions according to the day of the week. (TIF)

S1 Table Interventions for ischemic stroke. (DOCX)

S2 Table Interventions for hemorrhagic stroke. (DOCX)

S3 Table Threshold for annual intervention. (DOCX)

S4 Table Information on 70 hospital service areas. (DOCX)

S5 Table Seven parameters comprising the stroke severity index and associated explanations. (DOCX)

S6 Table Multiple linear regression model for evaluating the stroke severity index. (DOCX)

S7 Table Subgroup analysis for 30-day mortality according to admission on weekends among patients with hemorrhagic stroke. (DOCX)
