# Supplementary figures and images for: Weekend effect on 30-day mortality for ischemic and hemorrhagic stroke analyzed using severity index and staffing level
Source: PLoS One. 2023 Jun 22;18(6):e0283491. doi: 10.1371/journal.pone.0283491 (PMC10287008; doi:10.1371/journal.pone.0283491)

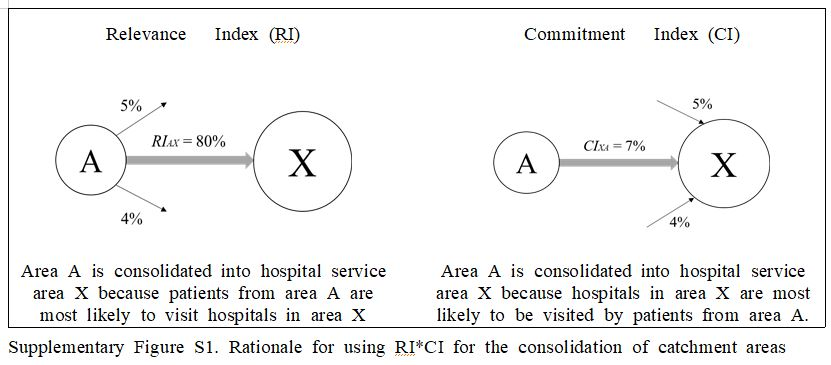

Supplement: S1 Fig — (TIF) [file pone.0283491.s001.tif]

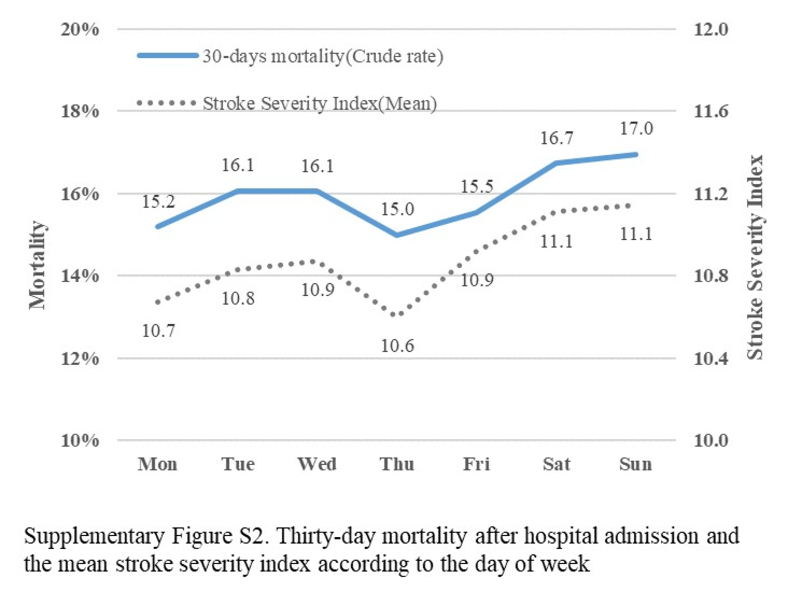

Supplement: S2 Fig — (TIF) [file pone.0283491.s002.tif]

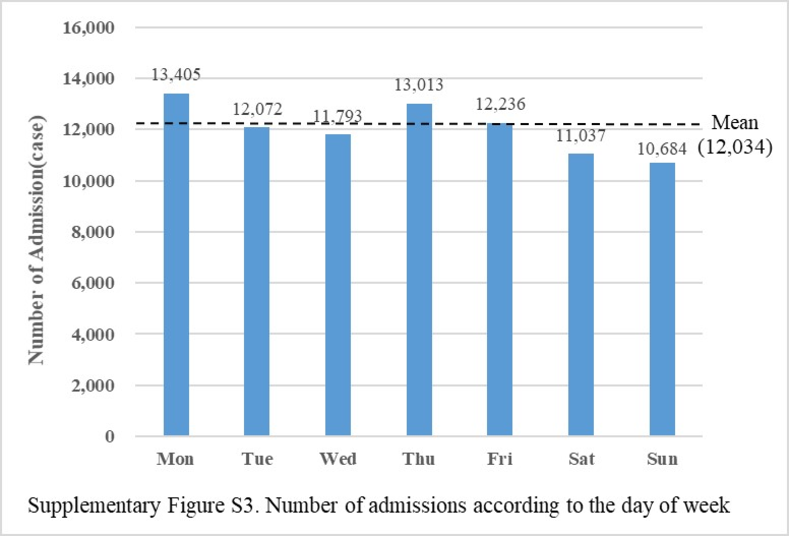

Supplement: S3 Fig — (TIF) [file pone.0283491.s003.tif]
