# Supplementary material for: Weekend effect on 30-day mortality for ischemic and hemorrhagic stroke analyzed using severity index and staffing level
Source: PLoS One. 2023 Jun 22;18(6):e0283491. doi: 10.1371/journal.pone.0283491 (PMC10287008; doi:10.1371/journal.pone.0283491)
Supplement: S1 Table — (DOCX) [file pone.0283491.s004.docx]

Supplementary Table S1. Interventions for ischemic stroke

| Type | Contents | EDI codes |
| --- | --- | --- |
| Medications[21] | rt-PA | 223501BIJ, 223502BIJ |
|  | New antithrombotics ≤3 days | Aspirin: 110701ATB, 110701ATE, 110702ATB, 110801ATB, 110802ATB, 111001ACE, 111001ACH, 111001ATB, 111001ATE, 111002ATE, 111003ACE, 111003ATE, 256800ATB, 517900ACE, 517900ATE, 517900ACH |
|  |  | Clopidogrel: 136901ATB, 492501ATB, 495201ATB, 498801ATB, 501501ATB, 517900ACE, 517900ATE, 517900ACH |
|  |  | Cilostazol: 133201ACR, 133201ATB, 133201ATR, 133201ATD, 133202ATB, 133203ATR, 506100ATB |
|  |  | Triflusal: 244101ACE, 244101ACH, 244102ACH |
|  |  | Ticlopidine: 239201ATB, 239202ATB, 498900ATB, 565300ATB |
|  |  | Warfarin: 249103ATB, 249105ATB, 249101ATB, 249102ATB, 249104ATB, 249106ATB, 249107ATB, 249108ATB, 249109ATB |
|  |  | Rivaroxaban: 511401ATB, 511402ATB, 511403ATB, 511404ATB |
|  |  | Dabigatran: 613701ACH, 613702ACH, 613703ACH |
|  |  | Apixaban: 617001ATB, 617002ATB |
|  |  | Edoxaban: 643601ATB, 643602ATB, 643603ATB |
|  |  | Enoxaparin: 152130BIJ, 152131BIJ, 152132BIJ, 152133BIJ, 152134BIJ, 152135BIJ, 152101BIJ, 152102BIJ, 152103BIJ, 152104BIJ, 152105BIJ, 152106BIJ |
|  |  | Dalteparin: 140230BIJ, 140231BIJ, 140232BIJ, 140233BIJ, 140234BIJ, 140201BIJ, 140202BIJ, 140203BIJ |
|  |  | Nadroparin: 198401BIJ, 198402BIJ, 198403BIJ, 198404BIJ, 198405BIJ, 198406BIJ, 198430BIJ, 198431BIJ, 198432BIJ |
|  |  | Heparin: 168630BIJ, 168632BIJ, 168631BIJ, 168601BIJ, 168602BIJ |
|  | Anticoagulants ≤7 days | Claims codes for warfarin, rivaroxaban, dabigatran, apixaban, edoxaban, enoxaparin, dalteparin, nadroparin, and heparin |
| Procedures | Percutaneous thrombus removal (thrombolysis): intracranial vessels, cerebral vessels, others[21] | M6630, M6631, M6632, M6633, M6635 |
|  | Percutaneous thrombus removal (mechanical thrombectomy): intracranial or extracranial vessels, others[21] | M6636, M6637, M6639 |
|  | CVAI[23] | M6594, M6602 |
|  | Intracranial artery angioplasty or stent insertion[23] | M6591, M6593, M6601, O1637 |
|  | Percutaneous transluminal angioplasty–cerebral, others[21] | M6593, M6597, M6599 |
|  | Percutaneous intravascular installation of a metallic stent: cerebral, others[21] | M6601, M6605 |
|  | Therapeutic hypothermia[23] | M5970 |
| Operations | CEA[23] | O0226, O0227, O2066 |
|  | Direct and indirect intracerebral artery anastomosis[23] | S4661, S4662 |
|  | Craniectomy or craniotomy[23]/clipping[22] | N0332, N0333, S4610, S4621, S4622, S4640-S4642 |

CEA, carotid endarterectomy; CVAI, carotid/vertebral artery angioplasty or stent insertion; EDI, electronic data interchange; rt-PA, recombinant tissue plasminogen activator
