# Supplementary material for: Weekend effect on 30-day mortality for ischemic and hemorrhagic stroke analyzed using severity index and staffing level
Source: PLoS One. 2023 Jun 22;18(6):e0283491. doi: 10.1371/journal.pone.0283491 (PMC10287008; doi:10.1371/journal.pone.0283491)
Supplement: S2 Table — (DOCX) [file pone.0283491.s005.docx]

Supplementary Table S2. Interventions for hemorrhagic stroke

| Type | Definition | EDI codes |
| --- | --- | --- |
| Procedures | Therapeutic hypothermia[23] | M5970 |
|  | Coiling[22] | M1661, M1662, and M6641 |
| Operations | Craniectomy or craniotomy[23]/clipping[22] | N0332, N0333, S4610, S4621, S4622, S4640-S4642 |

EDI, electronic data interchange
