# Supplementary material for: Weekend effect on 30-day mortality for ischemic and hemorrhagic stroke analyzed using severity index and staffing level
Source: PLoS One. 2023 Jun 22;18(6):e0283491. doi: 10.1371/journal.pone.0283491 (PMC10287008; doi:10.1371/journal.pone.0283491)
Supplement: S3 Table — (DOCX) [file pone.0283491.s006.docx]

Supplementary Table 3. The threshold for annual intervention

| Intervention type | Definition | EDI codes | Threshold |
| --- | --- | --- | --- |
| Procedures | Percutaneous thrombus removal (thrombolysis): intracranial vessels, cerebral vessels, others[21] | M6630, M6631, M6632, M6633, M6635 | 10[25] |
|  | Percutaneous thrombus removal (mechanical thrombectomy): intracranial or extracranial vessels, others[21] | M6636, M6637, M6639 | 29[24] |
|  | CVAI[23] | M6594, M6602 | 29[24] |
|  | Intracranial artery angioplasty or stent insertion[23] | M6591, M6593, M6601, O1637 | 29[24] |
|  | Coiling[22] | M1661, M1662, M6641 | 14[27] |
| - Operations | CEA[23] | O0226, O0227, O2066 | 93[28] |
|  | Direct and indirect intracerebral artery anastomosis[23] | S4661, S4662 | 20[30,31] |
|  | Craniectomy or craniotomy[23]/clipping[23] | N0332, N0333, S4610, S4621, S4622 / S4640-S4642 | 30[29,32,33] |

CEA, carotid endarterectomy; CVAI, carotid/vertebral artery angioplasty or stent insertion; EDI, electronic data interchange
