# Supplementary material for: Weekend effect on 30-day mortality for ischemic and hemorrhagic stroke analyzed using severity index and staffing level
Source: PLoS One. 2023 Jun 22;18(6):e0283491. doi: 10.1371/journal.pone.0283491 (PMC10287008; doi:10.1371/journal.pone.0283491)
Supplement: S4 Table — (DOCX) [file pone.0283491.s007.docx]

**Catchment areas**

The regional characteristics evaluated in this study included catchment areas, and evaluations of inappropriate initial use of stroke interventions by the catchment area were performed. Catchment areas were classified into large treatment zones (catchment areas for upper-level hospitals), intermediate treatment zones (catchment areas for hospitals with bed capacity), and small treatment zones (catchment areas for primary care facilities), depending on the medical service used by the patient.

Moreover, catchment areas were calculated according to regional affinity, which was defined as the relevance index (RI) * the commitment index (CI). Specifically, we used RI*CI as a condition for the consolidation of catchment areas in this study. This method was developed based on the Dartmouth Atlas of Health Care (https://www.dartmouthatlas.org/) conditions for consolidation. We then modified these conditions through in-depth discussions with experts to accurately reflect the healthcare status in Korea (S1 Figure).

The minimum constraints for defining catchment areas were as follows: (1) a minimum background population of 150,000, (2) a minimum RI of 40%, and (3) a standard distance covered in 60 minutes from the seed region. A total of 50 intermediate treatment zones were derived from the catchment areas as a result of this analysis. This figure was ultimately modified to 70 intermediate treatment zones in consideration of city boundaries and the opinion of experts in the local government.^43^ We used intermediate treatment zones in the analysis in order to determine the gap in the utilization of medical services between regions. The composition of hospital service areas, which are the base unit (zone) of this study, is described in S4 Table.

We note that the RI refers to the percentage of patients who were treated within their own hospital service area according to their provided address, whereas the CI refers to the percentage of patients who came from the area where the hospital is located among the total patients treated at that hospital.

| Supplementary Table S4. Information on 70 hospital service areas | | |
| --- | --- | --- |
| **Group** | **Service area** | **City (county, district)** |
| 1 | Northwest Seoul | Seoul (Jongno-gu, Jung-gu, Yongsan-gu, Seodaemun-gu, Mapo-gu, Eunpyeong-gu) |
| 2 | Northeast Seoul | Seoul (Dongdaemun-gu, Jungnang-gu, Seongdong-gu, Gwangjin-gu, Dobong-gu, Nowon-gu, Gangbuk-gu, Seongbuk-gu) |
| 3 | Southwest Seoul | Seoul (Yangcheon-gu, Guro-gu, Gangseo-gu, Geumcheon-gu, Yeongdeungpo-gu) |
| 4 | Southeast Seoul | Seoul (Seocho-gu, Gangnam-gu, Gangdong-gu, Songpa-gu, Gwanak-gu, Dongjak-gu) |
| 5 | West Busan | Busan (Gangseo-gu, Saha-gu, Sasang-gu, Buk-gu) |
| 6 | Mid-Busan | Busan (Seo-gu, Jung-gu, Dong-gu, Yeongdo-gu, Nam-gu, Busanjin-gu, Yeonje-gu, Dongnae-gu) |
| 7 | East Busan | Busan (Geumjeong-gu, Gijang-gun, Suyeong-gu, Haeundae-gu) |
| 8 | Northeast Dague | Daegu (Dong-gu, Buk-gu, Jung-gu, Suseong-gu) |
| 9 | Southwest Dague | Daegu (Nam-gu, Dalseo-gu, Dalseong-gun, Seo-gu) |
| 10 | Northwest Incheon | Incheon (Seo-gu, Ganghwa-gun) |
| 11 | Northeast Incheon | Incheon (Bupyeong-gu, Gyeyang-gu) |
| 12 | Mid-Incheon | Incheon (Jung-gu, Nam-gu, Dong-gu, Ongjin-gun) |
| 13 | South Incheon | Incheon (Yeonsu-gu, Namdong-gu) |
| 14 | West Gwangju | Gwangju (Gwangsan-gu, Seo-gu) |
| 15 | Southeast Gwangju | Gwangju (Buk-gu, Dong-gu, Nam-gu) |
| 16 | West Daejeon | Daejeon (Yuseong-gu, Seo-gu) |
| 17 | East Daejeon | Daejeon (Daedeok-gu, Dong-gu, Jung-gu) |
| 18 | Southwest Ulsan | Ulsan (Ulju-gun, Jung-gu) |
| 19 | Northeast Ulsan | Ulsan (Nam-gu, Buk-gu, Dong-gu) |
| 20 | Sejong-si | Sejong-si |
| 21 | Suwon-si | Gyeonggi-do (Suwon-si, Hwaseong-si, Osan-si) |
| 22 | Seongnam-si | Gyeonggi-do (Seongnam-si ,Yongin-si ,Gwangju-si, Hanam-si) |
| 23 | Uijeongbu-si | Gyeonggi-do (Uijeongbu-si, Dongducheon-si, Yangju-si, Yeoncheon-gun), |
| 24 | Anyang-si | Gyeonggi-do (Anyang-si, Gwacheon-si, Uiwang-si, Gunpo-si) |
| 25 | Bucheon-si | Gyeonggi-do (Bucheon-si, Gwangmyeong-si) |
| 26 | Pyeongtaek-si | Gyeonggi-do (Pyeongtaek-si, Anseong-si) |
| 27 | Ansan-si | Gyeonggi-do (Ansan-si, Siheung-si) |
| 28 | Goyang-si | Gyeonggi-do (Goyang-si, Gimpo-si) |
| 29 | Namyangju-si | Gyeonggi-do (Namyangju-si, Guri-si, Yangpyeong-gun, Gapyeong-gun) |
| 30 | Paju-si | Gyeonggi-do (Paju-si) |
| 31 | Icheon-si | Gyeonggi-do (Icheon-si, Yeoju-si) |
| 32 | Pocheon-si | Gyeonggi-do (Pocheon-si) |
| 33 | Chuncheon-si | Gangwon-do (Chuncheon-si, Hongcheon-gun, Hwacheon-gun, Yanggu-gun, Cheorwon-gun) |
| 34 | Wonju-si | Gangwon-do (Wonju-si, Hoengseong-gun) |
| 35 | Yeongwol-gun | Gangwon-do (Yeongwol-gun, Jeongseon-gun, Pyeongchang-gun) |
| 36 | Gangneung-si | Gangwon-do (Gangneung-si) |
| 37 | Donghae-si | Gangwon-do (Donghae-si, Taebaek-si, Samcheok-si), |
| 38 | Sokcho-si | Gangwon-do (Sokcho-si, Goseong-gun, Inje-gun, Yangyang-gun) |
| 39 | Cheongju-si | Chungcheongbuk-do (Cheongju-si, Jeungpyeong-gun , Jincheon-gun, Okcheon-gun , Yeongdong-gun , Boeun-gun) |
| 40 | Chungju-si | Chungcheongbuk-do (Chungju-si, Goesan-gun, Eumseong-gun) |
| 41 | Jecheon-si | Chungcheongbuk-do (Jecheon-si, Danyang-gun) |
| 42 | Cheonan-si | Chungcheongnam-do (Cheonan-si, Asan-si) |
| 43 | Gongju-si | Chungcheongnam-do (Gongju-si, Gyeryong-si) |
| 44 | Seosan-si | Chungcheongnam-do (Seosan-si, Taean-gun, Dangjin-si) |
| 45 | Nonsan-si | Chungcheongnam-do (Nonsan-si, Buyeo-gun, Geumsan-gun, Seocheon-gun) |
| 46 | Hongseong-gun | Chungcheongnam-do (Hongseong-gun , Cheongyang-gun, Yesan-gun, Boryeong-si) |
| 47 | Jeonju-si | Jeollabuk-do (Jeonju-si, Gimje-si, Wanju-gun, Jinan-gun, Muju-gun) |
| 48 | Gunsan-si | Jeollabuk-do (Gunsan-si) |
| 49 | Iksan-si | Jeollabuk-do (Iksan-si) |
| 50 | Jeongeup-si | Jeollabuk-do (Jeongeup-si, Gochang-gun, Buan-gun) |
| 51 | Namwon-si | Jeollabuk-do (Namwon-si, Sunchang-gun, Imsil-gun, Jangsu-gun) |
| 52 | Mokpo-si | Jeollanam-do (Mokpo-si, Yeongam-gun, Muan-gun, Sinan-gun, Jindo-gun, Hampyeong-gun) |
| 53 | Yeosu-si | Jeollanam-do (Yeosu-si) |
| 54 | Suncheon-si | Jeollanam-do (Suncheon-si, Gwangyang-si, Gurye-gun, Goheung-gun, Boseong-gun) |
| 55 | Naju-si | Jeollanam-do (Naju-si, Hwasun-gun, Gokseong-gun) |
| 56 | Haenam-gun | Jeollanam-do (Haenam-gun, Jangheung-gun, Gangjin-gun, Wando-gun) |
| 57 | Yeonggwang-gun | Jeollanam-do (Yeonggwang-gun, Damyang-gun, Jangseong-gun) |
| 58 | Pohang-si | Gyeongsangbuk-do (Pohang-si, Yeongdeok-gun, Uljin-gun, Ulleung-gun) |
| 59 | Gyeongju-si | Gyeongsangbuk-do (Gyeongju-si, Gyeongsan-si, Cheongdo-gun, Yeongcheon-si) |
| 60 | Andong-si | Gyeongsangbuk-do (Andong-si, Uiseong-gun, Cheongsong-gun, Yeongyang-gun) |
| 61 | Gumi-si | Gyeongsangbuk-do (Gumi-si, Chilgok-gun, Gunwi-gun, Gimcheon-si, Seongju-gun, Goryeong-gun) |
| 62 | Yeongju-si | Gyeongsangbuk-do (Yeongju-si, Yecheon-gun, Bonghwa-gun) |
| 63 | Sangju-si | Gyeongsangbuk-do (Sangju-si, Mungyeong-si) |
| 64 | Changwon-si | Gyeongsangnam-do (Changwon-si, Uiryeong-gun, Haman-gun, Changnyeong-gun) |
| 65 | Jinju-si | Gyeongsangnam-do (Jinju-si, Sancheong-gun, Hadong-gun, Sacheon-si, Namhae-gun) |
| 66 | Tongyeong-si | Gyeongsangnam-do (Tongyeong-si, Geoje-si, Goseong-gun) |
| 67 | Gimhae-si | Gyeongsangnam-do (Gimhae-si, Miryang-si, Yangsan-si) |
| 68 | Geochang-gun | Gyeongsangnam-do (Geochang-gun, Hapcheon-gun, Hamyang-gun) |
| 69 | Jeju-si | Jeju-si |
| 70 | Seogwipo-si | Seogwipo-si |
