# Supplementary material for: Weekend effect on 30-day mortality for ischemic and hemorrhagic stroke analyzed using severity index and staffing level
Source: PLoS One. 2023 Jun 22;18(6):e0283491. doi: 10.1371/journal.pone.0283491 (PMC10287008; doi:10.1371/journal.pone.0283491)
Supplement: S5 Table — (DOCX) [file pone.0283491.s008.docx]

| Supplementary Table S5. Seven parameters comprising the stroke severity index and associated explanations[20] | | |
| --- | --- | --- |
| Descriptions | Explanation | Code system / codes |
| Airway suctioning | Suctioning of a patient’s airway is undertaken in patients with an altered mental state or for those undergoing anesthesia during an ERT procedure | HIRA code / M0135, M0137 |
| Bacterial sensitivity test | Almost every patient receiving intravenous antibiotics has undergone this test | HIRA code / B40xx, B413xx, B414x |
| General ward stay | Relatively mild stroke patients admitted from the emergency department are usually first admitted to the general ward | HIRA code / ABxx |
| ICU stay | Severe stroke patients admitted from the emergency department are almost always first admitted to the ICU | HIRA code / AJxxx |
| Nasogastric intubation | Severe stroke patients cannot eat by themselves, and therefore, this tube is usually inserted to ensure proper nutrition | HIRA code / Q2621, Q2622 |
| Osmotherapy | For a severe stroke patient whose brain tissue is massively damaged, osmotherapy is usually prescribed to prevent brain herniation | Main component code /  14800Xbij, 148010BIJ, 148011BIJ |
| Urinary catheterization | Severe stroke patients cannot walk by themselves and therefore usually void through urinary catheterization | HIRA code / M0060 |
| ERT, endovascular recanalization therapy; HIRA, Health Insurance Review and Assessment Service; ICU, intensive care unit; “x” indicates various numbers (from 1 to 9) | | |
