# Supplementary material for: Weekend effect on 30-day mortality for ischemic and hemorrhagic stroke analyzed using severity index and staffing level
Source: PLoS One. 2023 Jun 22;18(6):e0283491. doi: 10.1371/journal.pone.0283491 (PMC10287008; doi:10.1371/journal.pone.0283491)
Supplement: S6 Table — (DOCX) [file pone.0283491.s009.docx]

Supplementary Table S6. Multiple linear regression model for evaluating the stroke severity index[20]

| Predictor | Coefficient |
| --- | --- |
| Airway suctioning | 3.7345 |
| Bacterial sensitivity test | 1.1990 |
| General ward stay | -2.9088 |
| ICU stay | -0.1125 |
| Nasogastric intubation | 3.4404 |
| Osmotherapy | 1.8908 |
| Urinary catheterization | 2.7366 |
| (Constant) | 5.8726 |
| ICU, intensive care unit | |
