# Supplementary material for: Weekend effect on 30-day mortality for ischemic and hemorrhagic stroke analyzed using severity index and staffing level
Source: PLoS One. 2023 Jun 22;18(6):e0283491. doi: 10.1371/journal.pone.0283491 (PMC10287008; doi:10.1371/journal.pone.0283491)
Supplement: S7 Table — (DOCX) [file pone.0283491.s010.docx]

Supplementary Table S7. Subgroup analysis for 30-day mortality according to admission on weekends among patients with hemorrhagic stroke

| Variable | | |  | 30-day mortality | | | |  |
| --- | --- | --- | --- | --- | --- | --- | --- | --- |
|  |  |  | Weekend | | | Weekday | | |
|  |  |  | OR | 95% CI | p-value | OR | 95% CI | p-value |
| **Patient-level** | | |  |  |  |  |  |  |
|  | Sex | |  |  |  |  |  |  |
|  | Male | | 1.00 |  |  | 1.00 |  |  |
|  | Female | | 1.008 | 0.92-1.11 | 0.8612 | 0.96 | 0.91-1.01 | 0.1198 |
|  | Age | | 1.02 | 1.02-1.02* | <0.0001 | 1.02 | 1.02-1.03* | <0.0001 |
|  | Income level | |  |  |  |  |  |  |
|  | Health insurance | 1^st^ quartile | 0.96 | 0.95-1.18 | 0.4444 | 0.95 | 0.89-1.01 | 0.0892 |
|  |  | 2^nd^ quartile | 1.06 | 0.95-1.18 | 0.2753 | 0.97 | 0.90-1.05 | 0.4731 |
|  |  | 3^rd^ quartile | 0.97 | 0.86-1.08 | 0.5229 | 1.00 | 0.94-1.06 | 0.8712 |
|  |  | 4^th^ quartile | 1.00 |  |  | 1.00 |  |  |
|  | Medical aid | | 1.17 | 1.00-1.38* | 0.0493 | 1.15 | 1.05-1.27* | 0.003 |
|  | SSI score | | 1.29 | 1.28-1.31* | <0.0001 | 1.29 | 1.28-1.30* | <0.0001 |
|  | Intervention: procedure | |  |  |  |  |  |  |
|  | Yes | | 1.00 |  |  | 1.00 |  |  |
|  | No | | 2.30 | 2.03-2.60* | <0.0001 | 1.83 | 1.69-1.99* | <0.0001 |
|  | Intervention: operation | |  |  |  |  |  |  |
|  | Yes | | 1.00 |  |  | 1.00 |  |  |
|  | No | | 2.25 | 1.98-2.56* | <0.0001 | 2.03 | 1.89-2.18* | <0.0001 |
| **Pre-hospital-level** | | |  |  |  |  |  |  |
|  | Type of contact with severe emergency center | | |  |  |  |  |  |
|  | Direct | | 1.00 |  |  | 1.00 |  |  |
|  | Transferring | | 1.36 | 1.06-1.75* | 0.0154 | 0.97 | 0.74-1.25 | 0.7919 |
| **Hospital level** | | |  |  |  |  |  |  |
|  | Type | |  |  |  |  |  |  |
|  | Tertiary hospital | | 1.00 |  |  | 1.00 |  |  |
|  | General hospital | | 0.86 | 0.75-0.98* | 0.0276 | 0.97 | 0.88-1.06 | 0.4718 |
|  | Hospital | | 2.43 | 1.60-3.69* | <0.0001 | 2.05 | 1.59-2.64* | <0.0001 |
|  | Bed volume | |  |  |  |  |  |  |
|  | ≤299 | | 0.93 | 0.59-1.45 | 0.733 | 1.24 | 0.87-1.75 | 0.2323 |
|  | 300-499 | | 0.93 | 0.65-1.32 | 0.6874 | 1.26 | 0.90-1.75 | 0.1763 |
|  | 500-999 | | 1.10 | 0.95-1.28 | 0.2149 | 1.23 | 1.04-1.44* | 0.0144 |
|  | ≥1000 | | 1.00 |  |  | 1.00 |  |  |
|  | Ownership | |  |  |  |  |  |  |
|  | Public | | 1.00 |  |  | 1.00 |  |  |
|  | Private | | 0.97 | 0.84-1.12 | 0.6705 | 1.00 | 0.89-1.11 | 0.9239 |
|  | Stroke center | |  |  |  |  |  |  |
|  | Yes | | 1.00 |  |  | 1.00 |  |  |
|  | No | | 1.05 | 0.94-1.18 | 0.3815 | 1.03 | 0.96-1.10 | 0.4836 |
|  | Intervention volume | |  |  |  |  |  |  |
|  | High-volume | | 1.00 |  |  | 1.00 |  |  |
|  | Low-volume | | 1.45 | 1.12-1.88* | 0.0047 | 1.42 | 1.24-1.63* | <0.0001 |
|  | Number of physicians | |  |  |  |  |  |  |
|  | 1^st^ quartile | | 1.20 | 0.93-1.57 | 0.1654 | 1.42 | 0.95-1.38 | 0.1679 |
|  | 2^nd^ quartile | | 1.19 | 1.00-1.41 | 0.0525 | 1.14 | 0.91-1.23 | 0.474 |
|  | 3^rd^ quartile | | 1.16 | 1.00-1.35 | 0.0508 | 1.06 | 0.93-1.19 | 0.4184 |
|  | 4^th^ quartile | | 1.00 |  |  | 1.00 |  |  |
|  | Number of nurses | |  |  |  |  |  |  |
|  | 1^st^ quartile | | 0.93 | 0.73-1.19 | 0.5607 | 0.92 | 0.74-1.15 | 0.4785 |
|  | 2^nd^ quartile | | 1.07 | 0.90-1.28 | 0.4567 | 0.94 | 0.82-1.07 | 0.329 |
|  | 3^rd^ quartile | | 1.12 | 0.97-1.28 | 0.1217 | 1.01 | 0.90-1.14 | 0.8844 |
|  | 4^th^ quartile | | 1.00 |  |  | 1.00 |  |  |
| CI, confidence interval; OR, odds ratio; SSI, stroke severity index  *p<0.05 | | | | | | | |  |
